# Mitochondrial ATP production is impaired in neural stem/progenitor cells derived from olfactory neuroepithelium of patients with schizophrenia

**DOI:** 10.1192/j.eurpsy.2021.1026

**Published:** 2021-08-13

**Authors:** C. Idotta, E. Tibaldi, N. Favaretto, M. Pagano, R. Peruzzo, G. Pigato, D. Cazzador, P. Meneguzzo, M. Solmi, L. Leanza, A. Favaro, A.M. Brunati, T. Toffanin

**Affiliations:** 1 Molecular Medicine, University of Padua, Padua, Italy; 2 Department Of Neuroscience, University of Padova, padova, Italy; 3 Biology, University of Padua, Padua, Italy; 4 Psychiatry Clinic, Azienda Ospedaliera di Padova, Padua, Italy; 5 Padua Neuroscience Center, University of Padua, Padua, Italy

**Keywords:** cellular models, mitochondria, schizophrénia, translational psychiatry

## Abstract

**Introduction:**

Neural stem/progenitor cells derived from olfactory neuroepithelium (hereafter OE-NS/PCs) are emerging as a viable proxy and a valuable tool for translational studies on severe mental illnesses (SMI). In this respect, the use of OE-NS/PCs as a surrogate cellular model of schizophrenia (SZ) has enabled insights into cell signaling and cell cycle dynamics in this disease.

**Objectives:**

We explored whether mitochondrial dysfunction, which has been already associated with SZ, may have a role in the altered proliferation pattern previously observed in OE-NS/PCs of SZ patients.

**Methods:**

OE-NS/PCs were collected from 20 patients and 20 healthy controls (Hcs) by nasal brushing, cultured in proper medium and expanded. Fresh OE-NS/PCs at passage 3 of both groups underwent BrdU proliferation assays or were frozen for later use. Mitochondrial ATP production was measured in both fresh and thawed OE-NS/PCs by using the ATPlite Luminescence Assay kit.

**Results:**

Fresh OE-NS/PCs of patients grew at a higher rate than those of HCs (M-W U=0; p<0.001), whereas the proliferation of thawed OE-NS/PCs of both groups exhibited an opposed pattern (at passage 6, p=0.002). Mitochondrial ATP production was significantly lower in OE-NS/PCs of patients than in those of HCs (M-W U=0; p=0.02), regardless of freeze-thaw conditions (M-W U=6; p=0.77).

**Conclusions:**

Mitochondrial ATP production is negatively affected in OE-NS/PCs of SZ patients as compared to those of HCs. This evidence does not differ in fresh OE-NS/PCs and OE-NS/PCs undergoing freeze-thaw cycles, which instead perturb the proliferation pattern of SZ OE-NS/PCs.

